# Non-clinical indicators of infertility and fertility care: a scoping review protocol

**DOI:** 10.1136/bmjopen-2025-115223

**Published:** 2026-05-28

**Authors:** Gitau Mburu, Luhua Zhao, Mei Mei, Peixuan Men, Jacky Boivin, James Kiarie

**Affiliations:** 1 UNDP-UNFPA-UNICEF-WHO-World Bank Special Programme of Research, Development and Research Training in Human Reproduction (HRP) Department of Sexual, Reproductive, Maternal, Child, Adolescent Health and Ageing, World Health Organization, Geneve, Switzerland; 2 Independent Consultant, Geneva, Switzerland; 3 Chinese Academy of Medical Sciences, Beijing, China; 4 Institute of Medical Information, Peking Union Medical College, Beijing, China; 5 Women’s Health Research Wales, School of Psychology, Cardiff University, Cardiff, UK

**Keywords:** PUBLIC HEALTH, Subfertility, Health Services, Systematic Review

## Abstract

**Abstract:**

**Introduction:**

Infertility affects millions of people globally. The presence of key indicators of infertility and fertility care is critical to monitoring access and utilisation of services as part of universal health coverage. In the area of infertility, it is important to track both clinical and non-clinical outcomes of care. Previous reviews have examined clinical indicators for infertility and fertility care; however, non-clinical indicators have received limited attention. This systematic scoping review aims to map the literature related to the non-clinical indicators of fertility care and their types and dimensions.

**Methods and analysis:**

This review will adhere to the established reporting guidelines for systematic scoping reviews and has been registered in the PROSPERO database to avoid duplication. A systematic literature search strategy will be developed and adapted to four electronic bibliographic databases (Medline [PubMed], EMBASE [Embase.com], CINAHL [EBSCOhost] and Web of Science [Clarivate]). Supplemental searches will be performed to obtain relevant grey literature from major health research institutes and international organisations. The search will be limited to literature published from 1990 to date. This review will cover all non-clinical interventions relevant to human infertility and fertility care. Two reviewers will independently screen papers and identify relevant studies for inclusion using predetermined criteria and extract data based on a predefined template. Types and dimensions of non-clinical indicators will be identified and a descriptive narrative synthesis will be used to describe the overall landscape. This review is expected to start on 1 June 2026 and conclude by 15 December 2026. A preliminary search strategy has been developed.

**Ethics and dissemination:**

As this study is based on a review of publicly available literature, ethical approval is not required. This scoping review will provide a comprehensive overview of the existing non-clinical indicators used to measure, report and monitor infertility and fertility care services. The findings will provide a comprehensive evidence base for developing a set of core indicators to monitor infertility and fertility care that can be adopted globally. Results will be disseminated through peer-reviewed publications, scientific conferences and webinars.

**PROSPERO registration number:**

CRD420251177901.

**STRENGTHS AND LIMITATIONS OF THIS STUDY**

- This review will synthesise diverse studies conducted using different methodologies to identify non-clinical indicators of infertility and fertility care globally.
- This review will not include an assessment of bias for the included studies.
- Some relevant studies may still be missed despite our planned systematic literature search strategy.

## Introduction

 Infertility is a disease of the reproductive system affecting both males and females, defined as the failure to achieve a pregnancy after 12 months or more of regular unprotected sexual intercourse.^1^ It is recognised as a global health issue, affecting up to 17.5% of adults of reproductive age during their lifetime.^2^ The prevalence of infertility does not vary significantly across regions or by income status of countries.^2^

The International Conference on Population and Development Programme of Action emphasises that the prevention and appropriate treatment of infertility should be integrated into the primary care services.^3^ Furthermore, the World Health Organization (WHO) underscores that providing high quality fertility care services is a core element of reproductive health^1^ and is essential for advancing progress towards Sustainable Development Goals (SDG) 3 and 5.^1 4^

Services for infertility encompass the prevention, diagnosis and treatment of infertility, covering a broad spectrum of interventions. Consequently, effective government policies are essential to ensure equitable access to appropriate fertility care as part of universal health coverage. In addition, it is crucial to establish a robust set of indicators that can measure the availability and utilisation of fertility care services and resources, as well as monitor the performance, affordability, quality and accessibility of fertility care.^5^ Indicators are markers of health status, service provision or resource availability, designed to enable the monitoring of service performance or programme goals.^5^ Consequently, the presence of key indicators of infertility and fertility care is critical towards monitoring progress as part of universal health coverage.

To inform the development of such an indicator set, WHO has conducted two key reviews, one on the prevalence of infertility and another on clinical indicators.^6 7^ These reviews have shown that existing clinical indicators primarily focus on in vitro fertilisation and other medically assisted reproduction services.^7^ However, the non-clinical indicators, which capture broader aspects of fertility care beyond clinical interventions, are largely unexplored. There is therefore a need to expand the potential pool of indicators that can be used as part of an indicator set for infertility and fertility care so as to include non-clinical indicators. In the area of infertility, it is important to track both clinical and non-clinical outcomes of care, as both can provide a more comprehensive picture of global response to infertility.

This scoping review aims to systematically identify, categorise, and propose a set of non-clinical indicators that can be used to monitor fertility care at global, regional and national levels. The review will provide an analysis of these indicators including their respective types and dimensions. The indicators that are anticipated to be identified through this review are expected to cover key domains such as health services (including availability, accessibility, affordability, health workforce, medicine and delivery), government policies and legal framework (eg, alternative therapies), social stigma and perception and psychosocial support.

Given the broad and multifaceted nature of non-clinical indicators related to fertility care, it is anticipated that the existing literature would encompass a diverse range of study designs and methodologies. Given this anticipated heterogeneity, a systematic scoping review is the most appropriate approach to map the breadth and extent of available evidence and synthesise existing non-clinical indicators that have been applied to measure and monitor fertility care. The findings will generate comprehensive evidence to inform future efforts in monitoring and improving fertility care services globally.

## Aim and objectives

The aim of this scoping review is to systematically examine non-clinical indicators that have been used to measure, report and monitor infertility and fertility care, and to synthesise the features of these indicators and the contexts in which they have been applied.

The specific objectives of the review are to:

1. Identify non-clinical indicators that have been used to measure, report and monitor infertility and fertility care in the published and grey literature.
2. Provide clear definitions and descriptions for identified indicators, including their intended purpose.
3. Map the identified indicators to their typologies, dimensions, measurement methodologies and levels of application in the health system.
4. Assess reported enablers, barriers and feasibility of collecting and deploying these indicators.
5. Propose a core set of potential non-clinical indicators that could be considered for routine measurement, reporting and monitoring of infertility and fertility care services.

## Research questions

This scoping review will be guided by the following research questions:

1. What non-clinical indicators have been used to measure, report and monitor fertility care?
2. How are these indicators defined, and what are their typologies and dimensions?
3. Which indicators are most commonly utilised, and what methodologies have been reported for their development, measurement and application?
4. At which levels of the health system (eg, national, subnational, facility) can these indicators be collected and monitored?
5. Which countries and regions have reported data on these indicators, and which organisations or agencies compile or maintain them?
6. What are the enabling factors and barriers affecting the collection, use and integration of these indicators into monitoring frameworks related to service provision (such as universal health coverage)?

## Materials and methods

### Study design

This review will be conducted in accordance with established methodological frameworks for scoping reviews proposed by Arksey and O’Malley.^8^ A scoping review is well suited for this kind of landscape analysis, particularly when a body of literature has not yet been comprehensively mapped or is expected to be heterogeneous in nature and therefore not amenable to a traditional systematic review.^9 10^ In addition, the review will adhere to the checklist of Preferred Reporting Items for Systematic Review and Meta-Analysis extension for scoping review (PRISMA).^11^

This protocol has been registered in the PROSPERO database, an international registry for systematic reviews (https://www.crd.york.ac.uk/PROSPERO, registration #: CRD420251177901), to minimise duplication and enhance transparency. Any amendments or deviations from this protocol will be clearly documented and justified in the final review report.

### Search strategy

A systematic literature search strategy will be developed in collaboration with an academic librarian, who will assist in constructing the initial search strings and refining search terms to ensure a comprehensive and accurate identification of relevant studies. The literature search will be conducted across several electronic bibliographic databases focusing on medical and health research, including Medline [PubMed], EMBASE [Embase.com], Cumulative Index of Nursing and Allied Health Literature (CINAHL) via EBSCOhost, and Web of Science [Clarivate].

To supplement the electronic database search, grey literature and reports will be retrieved through Google Scholar and online libraries of relevant organisations, including World Health Organization (WHO), United Nations Population Fund (UNFPA), International Committee for Monitoring Assisted Reproductive Technologies (ICMART), International Federation of Fertility Societies (IFFS), Fertility Europe and European Society of Human Reproductive and Embryology (ESHRE).

### Search terms

Search terms will include a combination of Medical Subject Headings (MeSH) and keywords related to infertility therapies, fertility care and non-clinical indicators. The search strings will be tailored to the indexing terms and syntax of each database. Please see online supplemental file 1 for provisional search terms.

### Eligibility criteria

To identify studies for inclusion, the review will follow a structured approach ensuring the inclusion is guided by the core concepts and research questions. The Sample, Phenomenon of interest, Design, Evaluation and Research type (SPIDER) framework will be applied to systematically guide the literature selection process.^12^ Table 1 shows a summary of the SPIDER we plan to use for the review.

**Table 1:** Inclusion and exclusion criteria

Concept	Inclusion	Exclusion
Sample	All population, particularly adults of reproductive age, health systems and services addressing infertility and fertility care.	Animal studies.
Phenomenon of interest	All non-clinical interventions or conditions relevant to infertility and fertility care.	Clinical interventions (eg, IVF success rate, laboratory procedures).
Design	Literature review	Not applicable.
Evaluation	All non-clinical indicators used to assess infertility and fertility care.	Studies focusing only on clinical indicators without any non-clinical components; duplicates or derivative analyses.
Research type	All study designs (observational, qualitative, quantitative, experimental and quasi-experimental) and relevant institutional reports and guidelines.	Expert opinions, editorials and literature reviews; retracted publications.
Time frame	Publications from 1 January 1990 to date to capture the recent and relevant evidence.	Studies whose primary data precede 1990, regardless of publication date.
Language	Non-English publications will be translated into English where feasible.	No restriction on publication language.

IVF, in vitro fertilisation.

### Screening procedure

Covidence will be used to manage the citations and remove the duplicates.^13^ Two reviewers will independently screen the titles, abstracts and full texts of studies for inclusion based on the predefined eligibility criteria. All discrepancies between the two reviewers will be resolved through discussion and consensus. If consensus cannot be reached, a third reviewer will be consulted to help finalise the decision. The study selection process and outcome will be presented using the PRISMA flow diagram.^11^

For non-English publications, titles and abstracts will first be translated into English using Google Translate during the initial screening. If additional information is needed to determine eligibility, the full text will also be translated into English. For studies that meet the inclusion criteria, the entire article will be translated to facilitate data extraction. When necessary and feasible, alternative translation tools will be employed to ensure accuracy.

The primary author of the publications may be contacted to validate the English translation.

### Data charting

A standardised electronic data extraction form will be developed and pilot-tested for use in this review. Two reviewers will independently extract data from all included studies. The extracted information will include bibliographic details such as author, year of publication, country and language; study characteristics including objectives, design and sample size; the indicators identified, along with their definitions, typologies and dimensions; the measurement methods, frequency of data collection, utility for economic or social evaluation and data sources used; any reported enablers and barriers; and the organisations or agencies responsible for the indicators.

### Quality assessment

Given the expected diversity in study design, methodological quality will be evaluated using the Integrated Quality Criteria for Review of Multiple Study Designs (ICROMS) tool.^14^ The ICROMS tool allows for the assessment of various study designs. It uses a ‘decision matrix’ and design-specific quality standards with a scoring system. Two reviewers will independently evaluate the methodological quality using the ICROMS criteria and scoring matrices. Scores produced by the two reviewers will be compared to ensure consistency. Any discrepancies in scoring will be resolved through consultation with a third reviewer.

### Data analysis and synthesis

Extracted data will be tabulated. The analysis will be primarily descriptive given the nature of a scoping review and the anticipated heterogeneity in the design of studies that will be included. The data synthesis will describe frequencies, themes and patterns observed across studies, and categorisation of indicators by their typologies, dimensions, level of application and measurement methodologies. Additionally, identified indicators will be mapped according to WHO regions, populations that the indicator applies to (eg, gender and age group) and other dimensions (such as patient-reported experience measures, eg, satisfaction, waiting times, accessibility and equity) to provide valuable insights for indicator selection and fertility care planning, implementation and monitoring.

This review is expected to start on 1 June 2026 and conclude by 15 December 2026. A preliminary search strategy has been developed.

## Dissemination

Results will be disseminated through peer-reviewed publications, scientific conferences and webinars.

### Strength and limitations

A key strength of this review is its ability to synthesise a body of diverse studies conducted using different methodologies across all global regions, thus providing a comprehensive and integrated overview of non-clinical indicators. Because of its broad scope, this review may identify gaps in the literature. A key limitation of this review is that it does not include an assessment of bias for the included studies. In addition, despite our planned broad and systematic literature search strategy, some relevant studies may still be missed. This scoping review aims to map out the existing universe of non-clinical indicators used. The findings will be more descriptive than definitive; they will not conclude the strength of these indicators and identify the most relevant or suitable ones. Thus, this review cannot answer specific clinical questions for informing clinical practices.

## Discussion

This scoping review will provide a comprehensive overview of the non-clinical indicators used to measure, report and monitor infertility and fertility care globally and its accessibility and equity. The review will support the engagement of a broad range of stakeholders to ensure that the findings are relevant and valid. The findings will support the development of a core indicator set to strengthen the monitoring framework for fertility care, inform policy-makers and health planners about existing indicators and gaps. The current effort will complement the existing clinical indicators identified. Together with clinical indicators, this will ultimately support the progress towards the achievement of SDGs 3 and 5.

## Supplementary material

10.1136/bmjopen-2025-115223 online supplemental file 1
