# Supplementary material for: Non-clinical indicators of infertility and fertility care: a scoping review protocol
Source: BMJ Open. 2026 May 28;16(5):e115223. doi: 10.1136/bmjopen-2025-115223 (PMC13223620; doi:10.1136/bmjopen-2025-115223)
Supplement: online supplemental file 1 [file bmjopen-16-5-s001.docx]

Search Strategy

# Overview

This scope review is a follow-up to a previous scope review that focused on clinical indicators of infertility and fertility care. The search strategy was developed around two core concepts as follows:

**Infertility and Fertility Care**

This concept excludes broader reproductive health areas, e.g., maternal care and contraception, that are not directly related to infertility and infertility cares.

**Non-Clinical Indicators**

This search focuses on the non-clinical indicators which refer to measures that do not directly assess the effectiveness or safety of medical interventions, including but not limited to:

- Equity: differences in access across administrative regions or socioeconomic groups;
- Patient-centeredness: patient satisfaction, psychosocial support, and informed consent;
- Timeliness: waiting times, and treatment intervals;
- Psychosocial: anxiety, depression, and family support;
- Access: geographic accessibility, financial affordability;
- Policy and ethics: legal or regulatory constraints, and ART ethical regulations;
- Economic burden: out-of-pocket costs, insurance reimbursement.

# Search Terms

## Infertility and Fertility Care

| Category | Terms and Variants |
| --- | --- |
| Infertility | Infertility; Subfertility*; Childlessness (involuntary); Fertility impairment |
| Care services | Fertility care; Fertility service; Infertility care; Reproductive care (for infertility) |
| Assisted reproduction | Assisted reproductive technology (ART); Medically assisted reproduction (MAR); ICSI; Embryo transfer (ET) |
| IVF | IVF; Fertilization in Vitro; Test-tube fertilization; Test tube babies |

## Non-Clinical Indicators

| Category | Terms and Variants | Excluded Clinical Terms |
| --- | --- | --- |
| Equity | Equity; Inequity; Disparity; Access gap; Socioeconomic difference; Geographic difference | - |
| Patient-centeredness | Patient-centeredness; Patient satisfaction; Patient experience; Psychosocial support; Shared decision-making | Patient outcome; Clinical satisfaction |
| Timeliness | Timeliness; Waiting time; Delay; Appointment interval; Treatment latency | - |
| Access | Access to fertility care; Geographic access; Financial access; Affordability; Insurance coverage | Service availability |
| Psychosocial | Psychosocial; Mental health; Anxiety; Depression; Stress; Family support; Stigma | Clinical psychology |
| Policy and ethics | Healthcare policy; Policy coverage; Ethics; Legal regulation | - |
| Economic burden | Economic burden; Cost; Out-of-pocket expenditure; Financial toxicity; Financial protection; Eligibility | Healthcare cost |

“Subfertility” is no longer a recommended term in research because of its poor definition. We decide to use it in our search as old articles may have used this term.

# Search Strategy Construction

1. **Medline (via Pubmed)**

Number of entries: 1634

Search structure: (Concept 1 terms) AND (Concept 2 terms) NOT (excluded clinical terms)

#1 "Infertility"[Mesh] OR "Subfertility"[tw] OR "Fertility Care"[tw] OR "Assisted Reproductive Technology"[Mesh] OR "Medically Assisted Reproduction"[tw] OR "Infertility Care"[tw] OR "Fertilization in Vitro"[Mesh] OR "In Vitro Fertilization"[tw] OR "Test-Tube Fertilization"[tw] OR "Test Tube Fertilization"[tw] OR "Test Tube Babies"[tw] OR IVF[tw]

#2 "Non-clinical Indicator"[tw] OR "Equity"[tw] OR "Patient-centeredness"[tw] OR "Timeliness"[tw] OR "Waiting Time"[tw] OR "Psychosocial"[tw] OR "Mental Health"[tw] OR "Access to Fertility Care"[tw] OR "Healthcare Policy"[tw] OR "Economic Burden"[tw] OR "Financial protection"[tw] OR "Eligibility"[tw] OR "Patient Satisfaction"[tw] OR "Fertility Care Disparity"[tw]

#3 "Effectiveness"[tw] OR "Efficiency"[tw] OR "Safety"[tw] OR "Pregnancy Rate"[tw] OR "Live Birth Rate"[tw] OR "Clinical Outcome"[tw]

#4 #1 AND #2 NOT #3

1. **Embase**

Number of entries: 6038

#1 'Infertility'/exp OR 'Assisted reproductive technology'/exp OR 'In vitro fertilization'/exp OR infertil*:ti,ab,kw OR subfertil*:ti,ab,kw OR "fertility care":ti,ab,kw OR "medically assisted reproduct*":ti,ab,kw OR "infertility care":ti,ab,kw OR "test-tube fertilization":ti,ab,kw OR "test tube fertilization":ti,ab,kw OR "test tube bab*":ti,ab,kw OR IVF:ti,ab,kw

#2 "non-clinical indicator*":ti,ab,kw OR equit*:ti,ab,kw OR "patient-centeredness":ti,ab,kw OR timeliness:ti,ab,kw OR "waiting time*":ti,ab,kw OR psychosocial:ti,ab,kw OR "mental health":ti,ab,kw OR "access to fertility care":ti,ab,kw OR "healthcare polic*":ti,ab,kw OR "economic burden*":ti,ab,kw OR "financial protection":ti,ab,kw OR eligibilit*:ti,ab,kw OR "patient satisfaction":ti,ab,kw OR "fertility care disparit*":ti,ab,kw

#3 effectiveness:ti,ab,kw OR efficienc*:ti,ab,kw OR safety:ti,ab,kw OR "pregnancy rate*":ti,ab,kw OR "live birth rate*":ti,ab,kw OR "clinical outcome*":ti,ab,kw

#4 (#1 AND #2) NOT #3

1. **CINAHL**

Number of entries: 3641

#1 MH="Infertility" OR "Assisted Reproductive Technology" OR "Fertilization in Vitro" OR MH="Subfertility" OR "Fertility Care" OR "Medically Assisted Reproduction" OR "Infertility Care" OR "In Vitro Fertilization" OR "Test-Tube Fertilization" OR "Test Tube Fertilization" OR "Test Tube Babies" OR "IVF"

#2 MH="Non-clinical Indicator" OR "Equity" OR "Patient-centeredness" OR "Timeliness" OR "Waiting Time" OR "Psychosocial" OR "Mental Health" OR "Access to Fertility Care" OR "Healthcare Policy" OR "Economic Burden" OR "Financial protection" OR "Eligibility" OR "Patient Satisfaction" OR "Fertility Care Disparity"

#3 MH=("Effectiveness" OR "Efficiency" OR "Safety" OR "Pregnancy Rate" OR "Live Birth Rate" OR "Clinical Outcome")

#4 #1 AND #2 NOT #3

1. **Web of Sciences**

Number of entries: 2265

#1 TS=(infertil* OR subfertil* OR "fertility care" OR "assisted reproduct* technolog*" OR "medically assisted reproduct*" OR "infertility care" OR "fertilization in vitro" OR "in vitro fertilization" OR "test-tube fertilization" OR "test tube fertilization" OR "test tube babies" OR IVF)

#2 TS=("non-clinical indicator*" OR equit* OR "patient-centeredness" OR timeliness OR "waiting time" OR psychosocial OR "mental health" OR "access to fertility care" OR "healthcare policy" OR "economic burden" OR "financial protection" OR eligibilit* OR "patient satisfaction" OR "fertility care disparit*")

#3 TS=(effectiveness OR efficienc* OR safety OR "pregnancy rate*" OR "live birth rate*" OR "clinical outcome*")

#4 (#1 AND #2) NOT #3

Unique entries from all searches: 6797

1. **Gray Literature search**

We will search for appropriate non-clinical indicators using a combination of the following key words: [infertility], AND [indicators] at the following websites:

1. World Health Organization: <https://iris.who.int/home>,
2. United Nation Population Fund (UNFPA): <https://www.unfpa.org/>,
3. International Committee for Monitoring Assisted Reproductive Technologies (ICMART): <https://www.icmartivf.org/>,
4. International Federation of Fertility Societies (IFFS): <https://www.iffsreproduction.org/>,
5. Fertility Europe and European Society of Human Reproductive and Embryology (ESHRE): <https://www.eshre.eu/>.
