# Supplementary material for: Non-clinical indicators of infertility and fertility care: a scoping review protocol
Source: BMJ Open. 2026 May 28;16(5):e115223. doi: 10.1136/bmjopen-2025-115223 (PMC13223620; doi:10.1136/bmjopen-2025-115223)
Supplement: Supplementary file 2 [file bmjopen-2025-115223.reviewer_comments.pdf]

PEER REVIEW HISTORY

BMJ Open publishes all reviews undertaken for accepted manuscripts. Reviewers are asked to complete a checklist review form and are provided with free text boxes to elaborate on their assessment. These free text comments are reproduced below.

ARTICLE DETAILS

Title (Provisional)

Non-Clinical Indicators of Infertility and Fertility Care – A Scoping Review Protocol

Authors

Mburu, Gitau; Zhao, Luhua; MEI, MEI; Men, Peixuan; Boivin, Jacky; Kiarie, James

---

VERSION 1 - REVIEW

**Reviewer**	**1**
**Name**	**January, James**
**Affiliation**	**University of Zimbabwe Faculty of Medicine and Health Sciences, Psychiatry**
**Date**	**02-Feb-2026**
**COI**	**None**

This is a good initiative and the authors are commended for proposing an appropriate design to broadly scope the topic. There are a few concerns which I have listed below:

1. The title mentions that this is a scoping review protocol, however the authors have listed “systematic review” as a key word. I would suggest that they use "systematic scoping review" as mentioned in their last paragraph before Aims.

2. In the keywords there is mention of “sub-fertility” which has not been defined in the manuscript although it is included among search terms.

3. In the abstract there is a statement which states that “This review … will be registered to avoid redundancy” yet there is already evidence that it was registered as stated in the main methods section (CRD 420251177901))

4. The statement, “Given the broad and multi-faceted nature of non-indicators…” should be revised to “Given the ….non-clinical indicators”

5. In the statement, “Additionally, identified indicators will be mapped …other dimensions (such as performance…”, please explain what is meant by performance.

6. Please include potential study limitations

7. There are several typos in the document which need correction, e.g., "…will be first translated into English using Google Translate During the initial screening" etc.

VERSION 1 - AUTHOR RESPONSE

Reviewer: 1

This is a good initiative and the authors are commended for proposing an appropriate design to broadly scope the topic.

Repones: Thank you!

There are a few concerns which I have listed below:

1. The title mentions that this is a scoping review protocol, however the authors have listed "systematic review" as a key word. I would suggest that they use "systematic scoping review" as mentioned in their last paragraph before Aims.

Response: We have changed it to "systematic scoping review" in both the title, keywords, and throughout the manuscript.

2. In the keywords there is mention of "sub-fertility" which has not been defined in the manuscript although it is included among search terms.

Response: We agree with the reviewer that sub-fertility is no longer a recommended term in research because of its poor definition. However, we used it in our search as old articles may have used this term.

We also added this clarification in the search strategy section to make it clear why it is used as a search term. In addition, it has been removed from the key word list.

3. In the abstract there is a statement which states that "This review … will be registered to avoid redundancy" yet there is already evidence that it was registered as stated in the main methods section (CRD 420251177901))

Response: We agree with the reviewer. The sentence now states "This review ... has been registered in PROSPERO database to avoid duplication".

4. The statement, "Given the broad and multi-faceted nature of non-indicators…" should be revised to "Given the ….non-clinical indicators"

Response: We agree with the reviewer, and it has been updated as suggested.

5. In the statement, "Additionally, identified indicators will be mapped …other dimensions (such as performance…", please explain what is meant by performance.

>> Thank you for pointing it out, we have deleted this term, and the sentence now states "(such as patient reported experience measures, e.g., satisfaction, waiting times, accessibility and equity)"

6. Please include potential study limitations

Response: We have inserted a new paragraph before discussion, highlighting the strength and limitations.

Strength and limitations

A key strength of this review is its ability to synthesize a body of diverse studies conducted using different methodologies across all global regions, thus providing a comprehensive and integrated overview of non-clinical indicators.

A key limitation of this review is that it does not include an assessment of bias for the included studies. In addition, despite our planned broad and systematic literature search strategy, some relevant studies may still be missed.

7. There are several typos in the document which need correction, e.g., "…will be first translated into English using Google Translate During the initial screening" etc.

Response: Thank you, we have carefully gone through the manuscript again, and have corrected the grammatical errors and typological mistakes.
